# Supplementary material for: Association of Food Allergy, Respiratory Allergy, and Skin Allergy with Attention Deficit/Hyperactivity Disorder among Children
Source: Nutrients. 2022 Jan 21;14(3):474. doi: 10.3390/nu14030474 (PMC8838767; doi:10.3390/nu14030474)
Supplement: Supplementary file 1 [file nutrients-14-00474-s001.zip › nutrients-1485137-supplementary.pdf]

Table S1. Characteristics of the participants by ADHD status.

Variables	Children without ADHD	Children with ADHD	
No. of participants	177197	15376	
Age, year	10.4 (0.01)	11.8 (0.04)	<.0001
Sex			
Boys	88142 (49.24%)	11011 (71.46%)	<0.001
Girls	89055 (50.76%)	4365 (28.54%)	
Race/ethnicity			
Hispanic	50166 (20.67%)	2614 (12.40%)	<0.001
Non-Hispanic White	85250 (57.26%)	9425 (67.65%)	
Non-Hispanic Black	27067 (14.27%)	2416 (14.43%)	
Other	14714 (7.80%)	921 (5.52%)	
Family highest education level			
Less than high school	38314 (18.92%)	2878 (17.25%)	<0.001
High school	23088 (12.91%)	2338 (15.32%)	
College or higher	114787 (67.66%)	10131 (67.25%)	
Missing	1008 (0.52%)	29 (0.18%)	
Family income to poverty ratio			
<1.0	25034 (14.12%)	2719 (18.31%)	<0.001
1.0-1.9	32379 (18.01%)	2996 (19.89%)	
2.0-3.9	45067 (25.88%)	3893 (25.19%)	
>=4.0	39358 (22.55%)	3468 (21.53%)	
Missing	35359 (19.43%)	2300 (15.08%)	
Geographic region			
Northeast	29812 (17.59%)	2539 (16.03%)	<0.001
Midwest	35939 (23.35%)	3561 (26.01%)	
South	63501 (35.67%)	6396 (42.16%)	
West	47945 (23.39%)	2880 (15.81%)	
Food allergy			
Yes	7565 (4.21%)	1038 (6.83%)	<0.001
No	169632 (95.79%)	14338 (93.17%)	
Respiratory allergy			
Yes	21380 (11.83%)	2838 (18.35%)	<0.001
No	155817 (88.17%)	12538 (81.65%)	
Skin allergy			
Yes	16604 (9.57%)	2099 (13.88%)	<0.001
No	160593 (90.43%)	13277 (86.12%)	

Data are presented as weighted means and standard errors in parentheses for continuous variables, and frequencies and weighted percentages in parentheses for categorical variables.

Table S2. Association of allergic conditions with ADHD, a sensitivity analysis by restricting to children (n=166,255) whose information was reported by their parents rather than other household members.

	Children without specific allergic conditions	Children with specific allergic conditions	P-value
**Food allergy**			
No. of ADHD cases/total participants	12268/158625	901/7630	
Model 1[a]	1.00 (reference)	**1.73 (1.55-1.93)**	**<0.001**
Model 2[b]	1.00 (reference)	**1.72 (1.55-1.92)**	**<0.001**
Model 3[c]	1.00 (reference)	**1.44(1.28-1.62)**	**<0.001**
**Respiratory allergy**			
No. of ADHD cases/total participants	10734/145176	2435/21079	
Model 1[a]	1.00 (reference)	**1.59 (1.49-1.69)**	**<0.001**
Model 2[b]	1.00 (reference)	**1.50 (1.41-1.60)**	**<0.001**
Model 3[c]	1.00 (reference)	**1.37 (1.28-1.46)**	**0.003**
**Skin allergy**			
No. of ADHD cases/total participants	11318/149557	1851/16698	
Model 1[a]	1.00 (reference)	**1.68 (1.57-1.79)**	**<0.001**
Model 2[b]	1.00 (reference)	**1.66 (1.55-1.77)**	**<0.001**
Model 3[c]	1.00 (reference)	**1.50 (1.39-1.61)**	**<0.001**

ADHD, attention deficit/hyperactivity disorder. [a] Model 1: adjusted for age and sex. [b] Model 2: model 1 plus race/ethnicity, family highest education level, family income to poverty ratio, and geographic region. [b] Model 3: model 2 plus mutual adjustment for other allergic conditions as mentioned.
